# Different Components of Sexual Narcissism Are Differentially Associated With Specific Sexual Aggression Strategies: An Exploratory Study Among Male and Female College Students

**DOI:** 10.1080/19317611.2024.2311142

**Published:** 2024-02-08

**Authors:** Maximiliane Uhlich, Zoë D. Peterson, Yi Li, Andrew Brown, Jin Han, Joseph A. D. McBride

**Affiliations:** a Department of Psychology, University of Basel, Basel, Switzerland; b Kinsey Institute, Indiana University, Bloomington, IN, USA; c Department of Counseling and Educational Psychology, Indiana University, Bloomington, IN, USA; d Department of Counseling, School, and Educational Psychology, University at Buffalo, Buffalo, NY, USA; e Department of Psychology, Jackson State University, Jackson, MS, USA

**Keywords:** Sexual Perpetration, Sexual Assault, Sexual Coercion, Sexual Narcissism, Sexual Aggression Strategies

## Abstract

**Objectives:**

This study investigated whether components of sexual narcissism are associated with different types of sexual aggression (e.g., verbal pressure or force) among students because this is a prevalent problem on college campuses.

**Methods:**

College students (N = 508) were recruited for a cross-sectional online study.

**Results:**

Results showed that components of sexual narcissism were related to different strategies of sexual aggression, with sexual entitlement being associated with not providing the victim an opportunity to object and exploiting intoxication. Gender moderated some of the associations, with women showing a stronger relationship than men.

**Conclusions:**

These findings suggest that sexual narcissism represents a risk factor for sexual aggression in men and women and highlight the need for gender-inclusive and tailored interventions to prevent sexual aggression.

Sexual aggression encompasses a diversity of behaviors and remains a prevalent problem. Commonly endorsed sexual aggression tactics include, use of verbal pressure and coercion, incapacitation from drugs or alcohol, physical force, or threats of physical violence to obtain sex from an unwilling partner (Peterson, 2023), and identifying risk factors for sexual aggression is important in order to effectively prevent it. In an early and classic study by Koss et al. (1987), 25% of college men reported having engaged in some type of sexual aggression toward women in a nationally representative sample, reflecting the diversity of higher education in the United States at the time. In that study, only men’s reports of perpetrating and women’s reports of experiencing sexual aggression were assessed (Koss et al., 1987). In years since, these results have been replicated in numerous studies, with approximately one third of male college students admitting to using verbally coercive strategies or taking advantage of situations when the victim was incapacitated in order to obtain sex (Abbey et al., 2001; DeGue & DiLillo, 2004; Zawacki et al., 2003). In addition to male college students engaging in sexual aggression, studies have found similar perpetration rates in community samples (Abbey et al., 2006; Senn et al., 2000), and approximately 15% of these incidents legally qualify as rape or attempted rape (Abbey et al., 1998).

For the purposes of this study, sexual aggression was broadly defined as obtaining oral, anal, or vaginal penetration (with a penis, finger, or other object) using strategies such as persisting after the person has refused (enticement); pressuring their partner into having sex with them (pressure); threats of harm or use of physical force (explicitly non-consensual); taking advantage of incapacitation due to alcohol or drugs (intoxication); and starting sex while a partner is asleep (asleep; Peterson, 2023). Being forced to penetrate someone else (with a penis, finger, or other object) was equally considered sexual aggression. These coercive strategies are based on the subscales of a newly developed measure, the Sexual Initiation Strategies Scale (SISS; Peterson, 2023), which was designed to assess sexual aggression perpetration.

### Male sexual aggression

In order to reduce the high prevalence of sexual aggression on college campuses (Duval et al., 2020) and to implement effective prevention programs is necessary. For instance, in a recent study conducted in the UK, 11.4% of participants self-reported sexual aggression (Hales & Gannon, 2022). Understanding and identifying risk factors for sexual aggression is crucial (Campbell et al., 2023; Erausquin et al., 2022) and will facilitate the development of interventions aimed at addressing and mitigating those specific risk factors. Notably, there may be different risk factors for different types of sexually aggressive strategies. For example, Lyndon et al. (2007) identified different risk factors for use of manipulation versus use of force to obtain sexual activity. In their study, men who reported using force were more likely to have experienced sexual abuse and witnessed domestic violence during childhood, more accepting of male violence, and less likely to endorse love as a motive for sex than men who reported having used manipulation or who reported no sexual aggression.

In a sample of incarcerated men, DeGue et al. (2010) discovered that men who engaged in nonphysical aggression (e.g., verbal pressure and manipulation) but not physical sexual aggression (e.g., incapacitation, physical force, or threats) were higher in the ability to manipulate others and to imagine others’ emotional reactions than physically sexually aggressive men. In contrast, men who engaged in physical sexual aggression were higher in egocentricity and hostility toward women than men who only engaged in nonphysical aggression. Other typical risk factors for male sexual aggression include atypical sexual fantasies, general aggression, hostility toward women, and rape myth acceptance (Hales & Gannon, 2022).

### Narcissism as a risk factor for male sexual aggression

One important predictor of sexual aggression among men is narcissism (Zeigler-Hill et al., 2013). Narcissism is characterized by a sense of grandiosity, need for admiration, sense of entitlement, lack of empathy, and a tendency to exploit others (American Psychiatric Association, 2000). This combination of low levels of empathy toward others (Watson et al., 1984), overestimation of skills, and the need for admiration (Morf & Rhodewalt, 2001) is problematic within the context of sexuality. For instance, men high in narcissistic traits tend to have more rape-supportive attitudes (Bushman et al., 2003) and are more likely to use various forms of pressure to obtain sexual contact (Kosson et al., 1997) than men low in narcissistic traits. Narcissism is also related to domestic violence (Simmons et al., 2005), sexual aggression (Mouilso & Calhoun, 2012), and family abuse (Day et al., 2022). In fact, sexual rejection may increase sexual desire in some narcissistic men, increasing their risk of sexual aggression (Bushman et al. 2003).

These studies have used a general scale to measure the construct of narcissism (e.g. Narcissistic Personality Inventory (NPI; Raskin & Terry, 1988). However, only certain aspects of general narcissism are related to sexual aggression (Zeigler-Hill et al., 2013). To account for this deficit, Widman and McNulty (2010) developed the more domain-specific Sexual Narcissism Scale (SNS) that identifies several components of sexual narcissism including sexual exploitation, sexual entitlement, low sexual empathy, and a grandiose sense of sexual skill. Hurlbert and Apt (1991) defined sexual narcissism earlier as an egocentric pattern of sexual behavior that manifests as being preoccupied with the satisfaction of one’s own needs rather than with those of their partner. The total score on the SNS has been shown to be related to rape myth acceptance (Peterson, 2023) and sexual aggression and to be a stronger predictor of men’s sexual aggression than general narcissism (Widman & McNulty, 2010). The four subscales of the SNS incorporate different facets of narcissism in a sexual context, shaping sexual behaviors and attitudes (Widman & McNulty, 2010). Sexual exploitation encompasses an individual’s willingness to manipulate a person to gain sexual activity. Sexual entitlement reflects an individual’s belief that they have the right to have their sexual needs fulfilled. Low sexual empathy refers to a lack of interest in the partner’s sexual experience. A grandiose sense of sexual skill represents an individual’s tendency to overestimate their sexual abilities. Widman and McNulty (2010) reported evidence that sexual narcissism is linked to various forms of sexual aggression among men, including engaging in unwanted sexual contact, verbal sexual coercion, and attempted or completed rape. Among the men in their sample, sexual aggression in general (including unwanted contact, verbal coercion, and attempted/completed rape) was also associated with each of the SN subscales but to different degrees. Starting with the strongest and ending with the weakest effect for the association between sexual aggression and SN, the following order emerged: sexual exploitation, sexual entitlement, low sexual empathy, and grandiose sense of sexual skill (Widman & McNulty, 2010). Recognizing individuals with an increased risk enables the targeting of prevention programs toward these specific individuals, and these findings suggest that men high in sexual narcissism—especially sexual exploitation and entitlement—are in particular need of intervention. The authors did not examine the relationship between the components of SN and different types of aggressive behaviors. Although there was a gender difference on SN scores, with men scoring higher than women, the authors did not examine the association between SN and sexual aggression among women.

### Female sexual aggression

Women are victims of sexual aggression more than men (Bhochhibhoya et al., 2021), and men are perpetrators more than women (Krahé & Berger, 2013; Struckman‐Johnson et al., 2003). Perhaps because of this, women’s sexual aggression has been far less studied than men’s, but research supports that women do use strategies of sexual aggression. Although women engage in sexual aggression less frequently than men (Buday & Peterson, 2015), in one study, 15% of women reported trying to get a man drunk and 6% reported using physical force to obtain sex (Anderson & Aymami, 1993). In another study, the most frequently endorsed sexual aggression strategy women used against men was verbal pressure and encouragement to drink alcohol (Struckman-Johnson & Struckman-Johnson, 1998). In a recent study, female sexual aggression was associated with lower scores on sexual assertiveness, higher scores on measures of acceptance of heterosexual male rape myths, early courtship rehearsal, and sexual sensation seeking (Struckman-Johnson et al., 2020). Buday and Peterson (2015) proposed that women’s unique sexually coercive strategies might not be captured in common self-report measures of sexual aggression. For example, in open-ended responses, they found that women reported using strategies that were not explicitly forceful. Instead, women simply did not provide an opportunity for their partner to refuse, including starting sex with a sleeping partner or starting the act without allowing their partner an opportunity to object.

Typically, prevention programs targeting actual or potential perpetrators are primarily directed toward or evaluated with men (DeGue et al., 2014). These previous findings underline the need for additional research on risk factors for women’s sexual aggression to reduce the prevalence of particular sexually aggressive strategies among both male and female perpetrators. Thus, prevention programs may benefit from incorporating strategies that target high risk women.

### Narcissism as a risk factor for female sexual aggression

Only a few studies have examined the relationship between women’s narcissism and sexual aggression. Although the literature supports the idea that women are, on average, less narcissistic than men (Grijalva et al., 2015), SN is a less gendered construct compared to other predictors of sexual aggression, such as rape myth acceptance or hostility toward women. This makes SN a more suitable predictor to explore among women and men. For instance, one study, using different measures, found general narcissism was associated with women’s sexual aggression (Blinkhorn et al., 2015). Specifically, in women, the entitlement/exploitativeness subscale of the NPI predicted use of sexual arousal strategies (e.g., persistently kissing and touching) after a partner said no, use of emotional manipulation, exploitation of intoxication, and use of physical force to obtain sex. In contrast, in men, the authority subscale of the NPI predicted emotional manipulation and exploitation of intoxication, with the subscale of grandiose/exhibitionism being related to sexual arousal strategies (Blinkhorn et al., 2015). Other studies have found that gender moderates the relationship between components of general narcissism and aggression in both in-person (Ménard & Pincus, 2022) and online contexts (March et al., 2021). For instance, in a laboratory study, women high in SN were less likely than men high in SN to use physical force in response to an experimental priming procedure known as priming sexual concepts (Imhoff et al., 2013). In another study, sexual aggression was associated with more socially toxic components of narcissism (i.e., entitlement/exploitativeness) for women, whereas it was associated with socially desirable aspects of narcissism (i.e., leadership/authority) for men (Blinkhorn et al., 2015).

With consideration of the reviewed literature, we expected gender to moderate the relationship between sexual narcissism and sexual aggression strategies, such that some aspects of sexual narcissism might predict certain sexually aggressive strategies differently for men compared to women. However, based on prior literature there is so far no evidence on specific predictors related to gender differently impacting the association between SN and sexually aggressive strategies. Thus, additionally to our main research question, we also examined gender as a potential moderator in an explorative manner. Understanding different predictors of sexual aggression among men and women and understanding specific correlates of different types of sexually aggressive strategies could allow interventionists to (1) identify and target individuals at high risk for perpetration, (2) tailor programs to be appropriate for both men and women, and (3) create programs that specifically address risk factors associated with different types of sexually aggressive strategies.

### The current study

This study included a sample of college students, as sexual aggression is a pervasive problem on university campuses due to the fact that college-age individuals are high-risk groups for sexual aggression (Muehlenhard et al., 2017). We explored whether different components of SN would predict different types of sexual aggression strategies. More specifically, we examined which components of SN independently predict each type of sexually aggressive strategy. Additionally, given that narcissism seems to present somewhat differently in men versus women, we examined gender as a moderator in the relationships between SN components and sexual aggression strategies.

## Method

### Participants

All participants were enrolled in a mid-sized, urban, Midwestern public university and recruited through a mass email. The students were invited to follow a link embedded in the invitation email to complete an online self-report study of “sexual behaviors and attitudes.” The student response was rapid, and data collection discontinued after three days due financial constraints. A total of 664 undergraduate and graduate students completed some portion of the questionnaire, and 575 completed the measures for this study. Participants were asked to indicate what their sex/gender was and were instructed to check all options that apply (male, female, intersex, gender queer, trans men, trans women). Because only ∼2% of participants identified with a gender other than male or female (10% did not specify their gender), too few to include them in the analysis, we decided to only include participants identifying as male or female in the analyses. The analyses and results reported here include a total of 508 individuals (144 men and 364 women of all sexual orientations), who completed at least 80% of the items on the measure of sexual narcissism, as that measure was the focus of this study. Participants were between 18 and 67 years old (*M*** **=** **24.89, *SD*** **=** **7.67), mostly heterosexual (∼77%) and predominantly White/European American (∼75%; see Table 1 for more demographic information). Every participant received a $10 online gift card in exchange for completing a 45-min online self-report measure. Students were informed that their privacy would be protected, and their name and email address were collected via a separate collector to be able to compensate participants afterwards, but identities could not be connected to their questionnaire responses. Participants did have the option to provide identifying information connected to their responses if they wanted to be contacted for additional studies on the same topic. The methods of this study were approved by the IRB at the relevant institution, and all students provided informed consent before participating in the study. The data reported here were collected as part of a larger measure validation study (Peterson, 2023). Table 2 displays the endorsement rates for each sexual aggression strategy.

**Table 1.** Sample demographics.

		Number of PP	Percentage of PP
Race	American Indian/Alaskan Native	8	1.6%
	Asian/Asian American	34	6.7%
	Native Hawaiian/Pacific Islander	3	0.6%
	Black/African American	73	14.3%
	White/European American	379	74.5%
	Hispanic/Latino/a	26	5.1%
	Other	20	3.9%
Relationship status	Single	304	59.7%
	Cohabitating	96	18.9%
	Married	79	15.5%
	Separated/divorced	9	1.8%
	Other	25	4.9%
Sexual identity	Heterosexual	390	76.6%
	Gay/Lesbian	20	3.9%
	Bisexual	51	10%
	Queer	8	1.6%
	Undecided	6	1.2%
	Other	15	2.9%

*Note. N* = 508.

**Table 2.** Number of participants endorsing different types of aggression strategies by gender.

		Men	Women
		N (%)	N (%)
Type of coercive strategy			
	Enticement	17 (11.8%)	35 (9.6%)
	Pressure	27 (18.8%)	36 (9.9%)
	No opportunity to object	23 (16%)	88 (24.2%)
	Intoxication	4 (2.8%)	4 (1.1%)
	Non-consensual	2 (1.4%)	–

*Note. N* = 144 men; *N* = 364 women.

### Measures

Sexual aggression was measured in the study using the new Sexual Initiation Strategies Scale (SISS; Peterson, 2023). Within the SISS, in order to track the gender of the sexual aggression victim, the participants were first asked, “With which gender do you typically engage in sexual activity? Based on whether participants selected “men” or “women,” they were directed to a version of the SISS with pronouns corresponding to that gender (if the participant has sex with both men and women, the answer was based on the gender that represents the majority of their recent sexual partners). Participants were asked to report on experiences with one gender because, for the larger study, we wanted to evaluate whether the measure was equally valid for reports of same- and other-gender perpetration (AUTHOR). Participants were asked to only report strategies that resulted in sex; thus, the SISS measures completed not attempted, sexual aggression. This decision was made because some sexually aggressive strategies do not seem compatible with attempted but not completed rape (see *Muehl*enhard et al., 2017, for a discussion). For example, it seems unlikely that someone would attempt but not succeed at having sex with someone who is too intoxicated to consent.

The SISS began with instructions stating:

We are interested in some of the common strategies that individuals use to initiate sex. Throughout this survey, when we say “sex,” we mean any of the following: Oral sex (one person’s mouth on another person’s genitals), Anal penetration (penetration of a person’s anus/butt with a penis, finger, or other object), Vaginal penetration (penetration of a woman’s vagina with a penis, finger, or other object). In the past, which if any of the following strategies have you personally used to get a woman/man to have sex with you? We are only interested in strategies that you used that actually resulted in oral sex, vaginal penetration, or anal penetration occurring.

Following the instructions, participants were presented with 39 coercive initiation strategies and 16 filler (noncoercive) initiation strategies and asked to check all strategies that apply.

Fornicola and Peterson (2022) identified four conceptual categories and multiple subcategories of tactics of the SISS for sexual aggression. In this study, we use their “broad categories of tactics: (1) *enticement* (three items, e.g. “After s/he initially says ‘no’ to sex, continuing to touch and kiss her/him in the hopes that s/he will give in to sex”), (2) *pressure* (21 items) with the sub-categories (a) situational pressure (e.g. “Taking her/him somewhere away from others and refusing to take her/him home unless s/he engages in sex”), (b) verbal pressure (e.g. “After s/he initially says ‘no’ to sex, reminding her/him that s/he owes you because of something non-sexual that you did”) and using (c) authority as pressure (e.g., “Using your older age to influence them to have sex”), (3) *ability to consent impaired* (eight items) with the subcategories (a) intoxication (e.g. “Taking advantage of the fact that s/he is too drunk or high”), (b) no opportunity to object (e.g., “Just starting the sexual act [e.g., while fooling around, penetrating their vagina or their anus when they weren’t expecting it] without providing an opportunity to object”), and (c) having sex with someone who is asleep (e.g., “Starting sex with them while they are asleep”), and (4) *explicitly nonconsensual* (seven items) with three subcategories (a) threats of harm, (b) use of force/restraint and (c) ignoring refusal. However, we examined two subcategories of Impaired Ability to Consent separately—Being impaired from alcohol or drugs and not being given the opportunity to object or asleep—because these two subcategories showed different patterns of results in Fornicola and Peterson’s (2022) study of sexual aggression as a function of victim and perpetrator gender, with the alcohol/drugs items being endorsed at the highest rates by men who have sex with men and the not being given the chance to object/asleep items being endorsed at the highest rate by women who have sex with men. This suggests that these subcategories may be utilized by different types of perpetrators and should perhaps be treated as conceptually distinct. These categories were conceptually-derived. We did not use exploratory factor analyses, nor do we report inter-item reliability for the subscales because the measure was conceptualized as a behavioral sampling measure, rather than a scale designed to assess a single latent construct (see Koss et al., 2007, for a discussion). Thus, *Koss* et al. (2007) advise against reporting internal reliability as this may not be the appropriate statistical method. Participants were classified dichotomously as having engaged or not engaged in each tactic of sexual aggression based on whether they endorsed one or more items consistent with that type of sexual aggression. Determining the frequency of sexual aggression retrospectively does usually not provide useful information due to social desirability and memory biases, especially in the area of sexuality (see e.g., Willis et al., 2021). Furthermore, as the distribution of these items is usually highly skewed (with only a minority admitting having engaged in sexual aggression), assessing sexual aggression dichotomously is typical for the field to measure aggressive behavior (see e.g., Postrefusal Sexual Persistence scale [PSP], Struckman‐Johnson et al., 2003).

In the validation study (see Peterson, 2023), the SISS demonstrated strong convergent validity based on its correlations with other relevant constructs and adequate test-retest reliability. Additionally, both male and female participants were more likely to endorse sexual aggression on the SISS than on two other measures of sexual aggression perpetration, suggesting that the SISS may capture instances of sexual aggression that were missed by other measures.

Participants also completed the Sexual Narcissism Scale (Widman & McNulty, 2010), which assesses components of SN on a scale from 1 (*Strongly Disagree*) to 5 (*Strongly Agree*). The scale has four subscales and captures the following different components: Sexual Exploitation (e.g., “One way to get a person in bed with me is to tell them what they want to hear”; in the current sample α = .75; *M*** **=** **1.51; *SD* = .24), Sexual Entitlement (e.g., “I am entitled to sex on a regular basis”; α = .76; *M*** **=** **1.70; *SD* = .27), Low Sexual Empathy (e.g., “The feelings of my sexual partners don’t usually concern me”; α = .68; *M*** **=** **1.50; *SD* = .21), and Grandiose Sense of Sexual Skill (e.g., “I am an exceptional sexual partner”; α = .88; *M*** **=** **3.45; *SD* = .15). Notably, the Low Sexual Empathy subscale demonstrated only marginally acceptable reliability in this study, and thus, results related to that subscale should be interpreted with caution. However, Widman and McNulty (2010) also report reliability values for alpha below .80 for sexual exploitation and low sexual empathy. All analyses were done using IBM SPSS Statistics (Version 28.0).

## Results

Table 3 displays the intercorrelations among the study variables, indicating the tactics on the SISS and the components of SN are almost all significantly correlated with each other.

**Table 3.** Biserial intercorrelations among the Sexual Narcissism Scale and Sexual Initiation Strategies Scale.

	1	2	3	4	5	6	7	8	9	10	11	12
1. Gender^a^	–											
2. Total SNS	−.14**	–			.							
3.SNS: sexual exploitation	−.21**	.83**	–									
4. SNS: sexual entitlement	−.06	.83**	.68**	–								
5. SNS: low sexual empathy	−.10*	.45**	.44**	.28**	–							
6. SNS: sexual skill	−.05	.57**	.21**	.27**	−.23**	–			.			
7. SISS: enticement^b^	−.03	.22**	.28**	.15**	.15**	.06	–		.		.	
8. SISS: pressure^b^	−.12**	.28**	.34**	.23**	.14**	.06	.33**	–				
9. SISS: no opportunity to object^b^	.09*	.24**	.21**	.23**	.08	.12**	.14**	.13**	–	.		
10. SISS: explicitly non-consensual^b^	−.10*	.17**	.17**	.17**	.01	.09*	.17**	.15**	.16**	–		
11. SISS: intoxication^b^	−.06	.15**	.11*	.16**	.04	.08	.17**	.19**	−.01	.15**	–	

*Notes.* Because individuals with missing data were excluded on a pairwise basis correlations range from 455 to 508. SNS: Sexual Narcissism Scale (Widman & McNulty, 2010); SISS: Sexual Initiation Strategies Scale (Peterson, 2023).

^a^ 0 = men and 1 = women.

^b^ 0 = no endorsement of items in that category and 1 = at least one item in that category endorsed.

*** *p* < .001; **p < .01; *p < .05.

To examine our research questions, we conducted a series of logistic regressions. First, a logistic regression was conducted with the four components of SN as the predictor variables and use of enticement strategies as the outcome variable (see Table 4). Results indicated that only sexual exploitation (*B*** ***=*** **1.32) was independently significantly (*p* < .001) associated with enticement strategies when all components of SN were entered into the model. The other components of SN were not significant in the model.

**Table 4.** Results of the four logistic regression analyses predicting four types of sexual aggression as a function of the components of sexual narcissism.

			Independent variables			
Dependent variables	Total *N* endorsement	Nagelkerke *R^2^*	Sexual exploitation *B(SE);* OR	Sexual entitlement *B(SE);* OR	Low sexual empathy *B(SE);* OR	Sexual skill *B(SE);*OR
Enticement	51	.14	1.32 (0.31); 3.73***	−0.45(0.27); 0.64	0.07(0.27); 1.07	0.07(0.20); 1.07
Pressure	61	.17	1.30(0.29); 3.65***	−0.02(0.24); 0.98	−0.20(0.27); 0.82	−0.09(0.18); 0.92
No opportunity to object	106	.09	0.30(0.23); 1.35	0.46(0.19); 1.58*	−0.04(0.23); 0.96	0.19(0.15); 1.21
Intoxication	8	.14	−0.18(0.56); 0.84	1.03(0.53); 2.81*	0.08(0.58); 1.08	0.53(0.52); 1.7

*Note. N* = 485.

*** *p* < .001; **p < .01; *p < .05.

Next, a logistic regression was conducted with the components of SN as the predictor variables and use of pressure as the outcome variable (see Table 4). Results indicated sexual exploitation (*B*** ***=*** **1.29) was again significantly (*p* < .001) and independently associated with the pressure strategies when all components of SN were entered into the model. The other components of SN were not significant.

In a next step, two separate logistic regressions were conducted with the components of SN as the predictor variables and with (1) no opportunity to object and (2) intoxication strategies as the outcome variables. Results indicated sexual entitlement was significantly and independently associated with both no opportunity to object (*B*** ***=*** **0.46, *p* = .015) and intoxication (*B*** ***=*** **1.03, *p* = .049) when all components of SN were entered into the model (see Table 4). The other components of SN were not significant. However, as only four individuals endorsed the intoxication strategy, this finding should be interpreted with caution.

We were unable to test if there was a significant relationship between the components of SN and an explicitly non-consensual strategy because too few individuals’ endorsement of use or threat of physical force (see Table 2).

**Figure 1.**
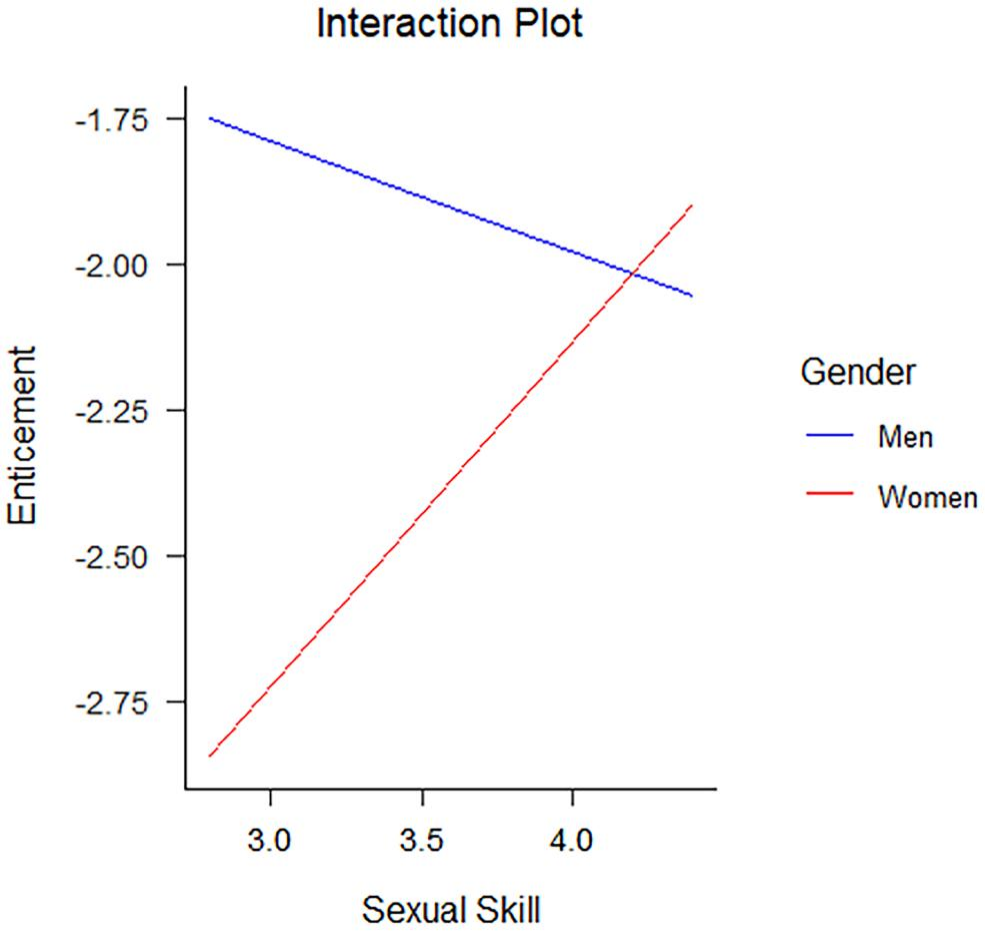
Significant interaction of grandiose sense of sexual skill by gender for use of enticement. The slope for women is significant; the slope for men is non-significant.

**Figure 2.**
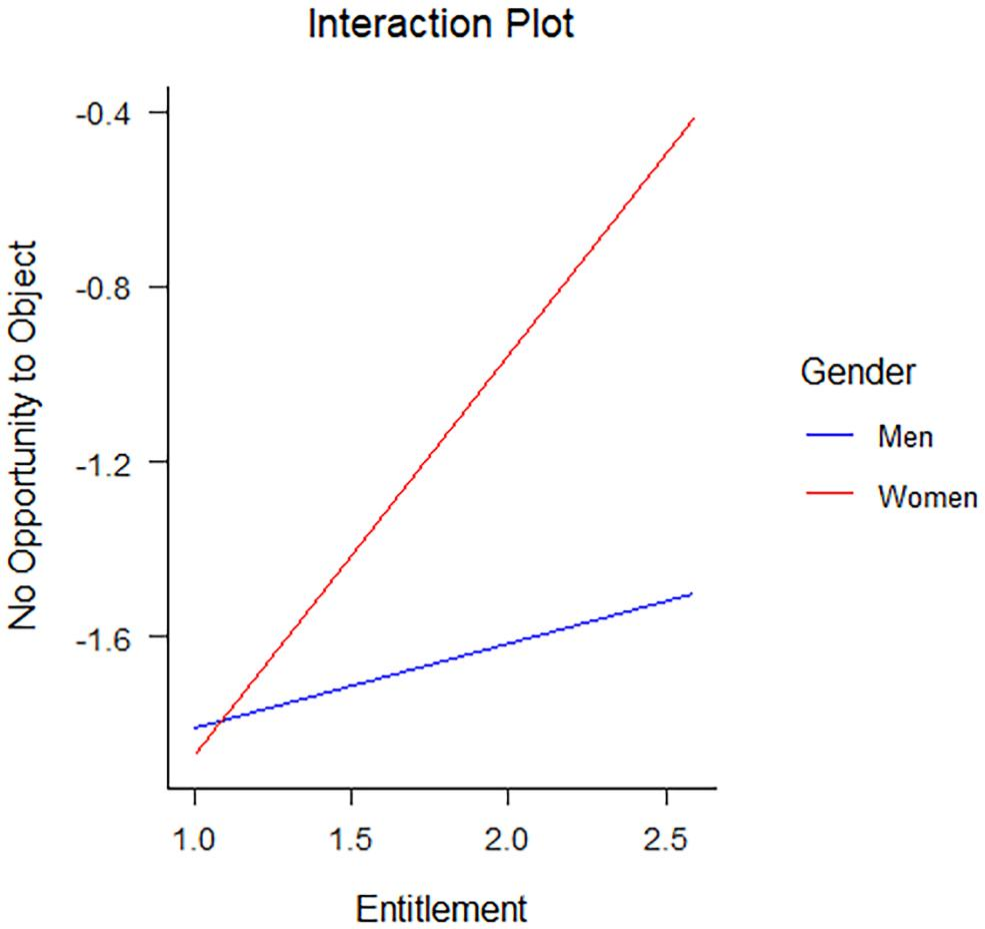
Significant interaction of sexual entitlement by gender for no opportunity to object. The slope for women is significant; the slope for men is non-significant.

To determine whether the relationship between SN and sexual aggression strategies varied as a function of gender, we tested gender as a moderator between each individual component of SN and sexual aggression strategy using the PROCESS macro (Hayes, 2012) for SPSS. The total sample size for this analysis was *N*** **=** **508. Using different sexual aggression strategies as dependent variables, we specified one model for every SN component and determined its interaction with gender (see Table 5 for all other non-significant interactions). The moderation analyses revealed a significant interaction between two components of SN and gender in predicting a sexually aggressive strategy. There was a significant interaction between grandiose sense of sexual skill and gender (*B* = .78, *SE* = .38, *p* .04) for use of enticement. The slope for women was significant and indicated a higher grandiose sense of sexual skill was associated with higher likelihood of endorsing use of enticement. For men, there was no significant relationship between grandiose sense of sexual skill and use of enticement (see Figure 1). Furthermore, there was a significant interaction between sexual entitlement and gender (*B* = .72, *SE* = .32, *p* = .02) for endorsement of no opportunity object. Again, the slope for women was significant with higher values of sexual entitlement being associated with a higher likelihood of engaging in sex without providing an opportunity to object. For men, the slope was not significant (see Figure 2). Although gender did not moderate the relationship between most components of SN and sexual aggression, our results revealed that gender did significantly moderate the relationship between two components of SN and sexual aggression strategies. In the two instances in which there was a significant moderation, the positive relationship between narcissism and likelihood of using sexual aggression strategies was greater for women than for men.

**Table 5.** Logistic regression models with gender as moderator.

	Dependent variables			
Independent variables	Enticement *B(SE)*	Pressure *B(SE)*	No opportunity to object *B(SE)*	Intoxication *B(SE)*
Sexual exploitation	0.61(0.26)*;	1.28(0.31)***;	0.48(0.25)	0.10(0.66)
Gender	−1.12(0.78)	0.49(0.82)	0.06(0.65)	−2.65(1.75)
Sexual exploitation × gender	0.54(0.37)	−0.39(0.39)	0.42(0.33)	1.13(0.80)
Nagelkerke *R^2^*	.12	.16	.09	.10
Sexual entitlement	0.10(0.30);	1.01(0.27)**	0.19(0.27)	0.40(0.56)
Gender	−1.41(0.79)	0.71(0.79)	−0.79(0.66)	−4.09(2.33)
Sexual entitlement × gender	0.53(0.38)	−0.63(0.36)	0.72(0.32)*	1.42(0.83)
Nagelkerke *R^2^*	.04	.09	.11	.17
Low sexual empathy	0.83(0.37)*;	1.10(36)**;	0.82(0.35)*	−0.55(1.20);
Gender	0.25(0.86)	0.83(0.83)	1.62(0.74)*	−2.97(2.13)
Low sexual empathy × gender	−0.36(0.46)	−0.84(0.46)	−0.65(0.41)	1.47(1.32)
Nagelkerke *R^2^*	.04	.06	.03	.05
Sexual skill	−0.19(0.28);	−0.04(0.25)	0.34(0.27)	0.48(0.69)
Gender	−3.28 (1.41)*	−2.16(1.28)	0.33(1.22)	−4.87(4.40)
Sexual skill × gender	0.78(0.38)*	0.43(0.35)	0.06(0.32)	1.06(1.05)
Nagelkerke *R^2^*	.04	.03	.04	.09

*Note. N_Empathy_* = 455; *N_Entitlement/Exploitation_* = 456; *N_Sexual skill_* = 454.

*** *p* < .001; **p < .01; *p < .05. Logistic regression coefficients for explicitly non-consensual are not displayed in the table due to low endorsement of those strategies.

## Discussion

Our results support prior research demonstrating that SN is associated with sexual aggression, and our results further demonstrate that different components of SN are associated with different types of sexual aggression. This replicates and extends work conducted by Widman and McNulty (2010), which established SN as an important correlate of sexual aggression perpetration in men. In our study, we found that sexual exploitation was a predictor for use of enticement and pressure, and sexual entitlement was a predictor for not providing an opportunity to object and intoxication strategies. Due to low base rates, we were not able to adequately examine the relationship between the components of SN and use of explicitly non-consensual strategies (e.g., force). Our results suggest that two components of SN—sexual exploitation and sexual entitlement—represent risk factors for different types of sexual aggression strategies. This supports previous work indicating the SN subscales of sexual exploitation and entitlement of SN are the components most strongly related to sexual aggression (Widman & McNulty, 2010). Indeed, sexual exploitation represents a willingness to engage in manipulation to obtain sex, and consistent with that, enticement and pressure both involve the use of pressure, manipulation, or continued persistence in the face of sexual rejection. Sexual entitlement involves the belief that one is owed or deserves sex whenever one wishes, and consistent with that perspective, sex without consent or with an intoxicated victim involves simply “taking” sex without the other person’s agreement. Thus, the relationship between these components of SN and these sexual aggression tactics makes intuitive sense.

This study also extends the work of Widman and McNulty (2010) by examining the components of SN as a risk factor for men’s and women’s sexual aggression. Female perpetrators are understudied, and our results support existing evidence (Buday & Peterson, 2015) of women’s sexual aggression. Our results also provided unique insight into particular risk factors for female sexual aggression. Although there were few differences between men and women in the components of SN and sexual aggression strategies (meaning that sexual narcissism—especially, sexual exploitation and entitlement—were risk factors for both men and women), our research revealed that gender did significantly moderate the relationship between SN and sexual aggression in two instances. These results suggest that some components of sexual narcissism were a more important predictor of women’s sexual aggression than men’s. Specifically, women who perceive themselves as highly skilled sexually are more likely than women who do not perceive themselves as highly skilled sexually to engage in enticement strategies to obtain sex. Additionally, women who feel more entitled to sex were more likely to engage in sex without explicit consent than women who felt less entitled. It is not clear why these relationships were significant for women not for men. One possible explanation might involve stereotypes of men’s sexuality. Stereotypically, men are expected to always be in the mood for sex; thus, men’s sexual consent may be perceived as irrelevant, as it can simply be assumed (Fornicola & Peterson, 2022; Peterson, 2018). Given that, heterosexual women who feel confident about their sexual skill and entitled to sexual pleasure may assume it is fine to persist in the face of sexual refusal and to move ahead without consent because men always want sex. Simultaneously, heterosexual men may be somewhat more inhibited to say “no” due to this stereotype. Few studies have evaluated risk factors involved in women’s sexual aggression, and our findings suggest components of SN are a strong correlate of women’s and men’s sexual aggression—and in two instances, a significant predictor only for women. Our results provide evidence that there is a need for gender specific interventions to reduce sexual aggression. Further research is needed on the development and implementation of successful gender-inclusive interventions and treatment programs aimed at identifying individuals fostering harmful attitudes to reduce sexually aggressive behaviors.

In our study, for both men and women, the SN components sexual exploitation and entitlement predicted several strategies of sexual aggression, suggesting these SN components represent similar risk factors for sexual aggression in women and men. These results may aid in characterizing and identifying high risk individuals and encourage the development of tailored prevention programs targeting specific risk factors to diminish rates of these specific strategies of sexual aggression among male and female perpetrators. Prevention programs aimed at actual or potential perpetrators are usually administered to and/or evaluated with men (DeGue et al., 2014), but our work suggests that including high risk women (e.g., sexually entitled women) in prevention efforts could be one promising avenue for reducing rates of sexual aggression among college students. Thus, prevention programs may benefit from incorporating prevention strategies that target unique risk factors and address the differential components of male and female sexual aggression.

So far, findings on the efficacy of sexual aggression prevention aimed at altering the underlying belief systems of potential perpetrators are discouraging, as most current programs yield only short-term effects in reducing rape-supportive attitudes and remain inadequate in lowering the prevalence of sexually aggressive behaviors (Wright et al., 2020). A clear description of what constitutes a high-risk population can aid in effectively distributing limited resources for prevention programs. One reason for the poor effects of existing prevention programs (DeGue et al., 2014) may be due to the lack of inclusion of highest risk individuals—those with a high likelihood of perpetration. Thus, our research suggests targeting efforts toward examining high-risk individuals is critically needed. Prevention models expanding their efforts to incorporate unique risk factors such as SN, which is linked to different types of sexual aggression, may help to improve the efficacy of prevention interventions.

### Limitations, research and prevention implications

There are several limitations of the present study. First, our study used a cross-sectional design and included self-report measures on sexual aggression strategies from a sample of mostly White, heterosexual college students, identifying as male or female. Thus, our results may not generalize to other populations, such as sexual and gender minorities. For participants indicating having sex with men and women, sexual aggression referred to the gender that represents the majority of their recent sexual partners. This potentially excluded previous accounts of possible sexual aggression with minority sexual partners.

Furthermore, this study measured only completed sexual aggression strategies, not attempted sexual perpetration. We made this decision because some sexual tactics do not seem entirely compatible with attempted but not completed sexual aggression (e.g., *Muehl*enhard et al., 2017). Furthermore, prior research has found that rates of attempted sexual assault are usually quite low, and most participants who report attempted sexual aggression perpetration also report completed sexual aggression perpetration (see Peterson, 2022, for a discussion). Nevertheless, future research could examine whether these relationships differ for individuals who have engaged in attempted and completed sexual aggression.

Additionally, the use of self-report methods for research on sexual aggression raises concerns about the role of socially desirability in participant responding (see Strang & Peterson, 2016). This might be especially true for college students who participate in mandatory trainings about what behaviors constitute as sexual assault when entering college (Katz & Moore, 2013). College students might be particularly aware of what behaviors are classified as sexual assault, and they may be less likely to admit to engaging in sexually aggressive behaviors than the general population. We may have encountered potential problems related to social desirability in this study, as reflected in the low frequencies of individuals who reported using physical force as an aggressive strategy.

Due to the low rates of endorsement of using physical force in our sample, we were not able to perform statistical analysis and determine the correlates of this type of coercive behavior. Relatedly, our finding regarding sexual entitlement predicting intoxication should be interpreted with caution as also only four participants endorsed that strategy. Also due to insufficient statistical power we did not examine interaction effects between SN and the gender of the *victim* to predict endorsements of different types of aggressive strategies. However, another study (Fornicola & Peterson, 2022) found that strategies of sexual aggression vary as a function of both the perpetrator’s and victim’s gender. Considering these findings, future research might benefit from investigating the interactions between SN and both the victim and perpetrator gender variables.

Future research might examine other components of the dark triad in predicting different types of sexual aggression perpetration and might consider gender as a moderator in that relationship. In prior research on the dark triad of personality traits, which includes psychopathy and Machiavellianism as well as narcissism, psychopathy was a significant predictor of sexual harassment victim-blaming among women and narcissism was not (Brewer et al., 2021). It is possible that other aspects of the dark triad predict use of sexual aggression more strongly than SN.

Another future research endeavor that might provide valuable insight would be the evaluation of the stability of SN components over time and the feasibility of changing SN beliefs through treatment or prevention interventions. We have argued here that it is important to understand the relationship between the components of SN and strategies of sexual aggression. Elucidating the effects of complex combinations of SN and sexual aggression strategies can inform prevention and treatment efforts; however, there is evidence that the general personality trait of narcissism is relatively stable across time (Edelstein et al., 2012) and thus may not serve as a promising point of intervention. Nevertheless, it is not clear to what extent that is true for SN, specifically. For example, future research could investigate the inter-individual variability of success rates aimed at reducing SN beliefs through preventative intervention efforts. Another example of a research design would be to determine whether or not SN can be reduced through interventions focused on sexual ethics training (see e.g., Lamb & Randazzo, 2016)—perhaps especially sexual ethics training that is delivered early (e.g., in middle school) before adolescents have solidified their attitudes toward dating and engaging in sexual behavior. Research could also investigate whether therapeutic interventions can successfully reduce sexual entitlement and exploitation in individuals who have already engaged in sexual aggression. Berg et al. (2017) provide one possible intervention model that could be evaluated to determine the efficacy in sexually aggressive individuals. Our research suggests future directions in this area of research are needed to determine if components of SN linked to coercive behaviors are modifiable and determine the impact of interventions aimed at reducing SN in potential or past sexual aggressors. Identifying attitudes and beliefs related to particular sexual aggression strategies could be promising to decrease sexual aggression and lower sexual perpetration rates among both men and women.

## Conclusions

Sexual aggression remains a prevalent problem among young adults and causes substantial suffering for victims with negative consequences for their mental and physical well-being (e.g. Jordan et al., 2010). This study identified sexual narcissism as an important risk factor for specific strategies of sexual aggression. Furthermore, gender moderated the association between some components of SN and sexually aggressive strategies. There are three important implications of this work: First, assessing the level of sexual narcissism in studies of sexual aggression may be beneficial as it constitutes an important risk factor for sexual aggression. Second, although rates of sexual aggression among women are lower than men, identifying high risk women and including them in prevention programs could improve the efficacy of these programs. Third and finally, being able to characterize the target population of sexual perpetrators will potentially help in developing interventions that reduce the overall rates of sexual aggression.

## Data Availability

The University of Missouri-St. Louis’ Institutional Review Board granted approval for the study methodologies detailed in this article.
